# Exogenous Glutathione and Nitric Oxide Improve Waterlogging Stress Tolerance in Maize

**DOI:** 10.1002/pei3.70136

**Published:** 2026-03-05

**Authors:** Prodipto Bishnu Angon, Md. Tahjib‐Ul‐Arif, Md. Sarwar Jahan, Md. Mahadi Hasan, Yoshiyuki Murata

**Affiliations:** ^1^ Applied Plant Biology and Bioinformatics Lab, Department of Biochemistry and Molecular Biology Bangladesh Agricultural University Mymensingh Bangladesh; ^2^ Department of Biochemistry and Molecular Biology Bangladesh Agricultural University Mymensingh Bangladesh; ^3^ Basic and Applied Scientific Research Centre Imam Abdulrahman Bin Faisal University Dammam Saudi Arabia; ^4^ Graduate School of Environmental and Life Science Okayama University Okayama Japan

**Keywords:** crop improvement, glutathione, maize, nitric oxide, stress tolerance

## Abstract

Maize ( *Zea mays* L.) is one of the major grain crops worldwide that is particularly vulnerable to waterlogging (WL) stress. Glutathione (GSH) and nitric oxide (NO) are known to protect plants from a variety of abiotic stresses; however, their potential for improving WL tolerance in maize remains unexplored. The present study examined the impact of exogenously applied GSH and NO on maize plants exposed to WL‐stress. Our findings revealed that GSH + NO‐treated waterlogged maize plants grew better and produced more biomass than only WL‐stressed plants. The improved performance of GSH + NO‐sprayed WL‐stressed maize seedlings was supported by the increased root dry and fresh weight, shoot length, shoot dry and fresh weight, chlorophyll *a*, chlorophyll *b*, and carotenoid content. Exogenous GSH and NO treatments significantly enhanced the amounts of leaf proline, leaf soluble sugars, and total protein in maize seedlings, suggesting adaptive metabolic reprogramming under stress. The increased malondialdehyde (MDA) levels and accumulation of hydrogen peroxide (H_2_O_2_) in maize leaves and roots revealed that WL caused significant oxidative damage. Exogenous GSH, NO individually, and combinedly significantly decreased total H_2_O_2_ and MDA contents in both leaves and roots. Exogenous GSH and NO reduced oxidative stress by increasing peroxidase activity, ascorbic acid, and anthocyanin content in maize leaf and root tissues. Our findings emphasize the possible relevance of GSH and NO, simultaneously and individually, in enhancing adaptive mechanisms in maize seedlings for reducing WL‐induced damage. Although the GSH + NO‐mediated approach shows promise for mitigating WL‐stress in maize under controlled conditions, further field‐based investigations are required to validate its practical applicability.

## Graphical abstract

Exogenous glutathione (GSH) and nitric oxide (NO) enhance waterlogging tolerance in maize seedlings by reducing oxidative damage and improving growth. Combined GSH + NO treatment reduces H_2_O_2_ and malondialdehyde levels, enhances antioxidant defenses, protects photosynthetic pigments, and promotes biomass accumulation, thereby improving the physiological performance of waterlogged maize seedlings.

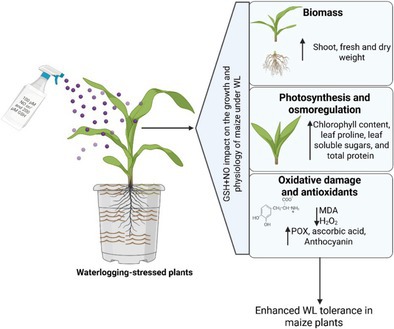

## 1 Introduction

Climate change is a common occurrence; however, accelerated climate change leads to abiotic challenges, such as water scarcity, waterlogging (WL), salinity, drought, and extreme temperatures for plants (Rhaman et al. 2024). Abiotic stress reduces crop yield by adversely influencing its internal metabolism, tissue growth, and development (Kumar 2020). WL is one of the most important abiotic stresses, causing significant crop yield loss worldwide (Fukao et al. 2019). Under WL stress, the soil surrounding plant roots becomes saturated with water, which lowers oxygen levels because water has a restricted ability to diffuse gases (Xu et al. 2016; Ren et al. 2017).

Roughly 10%–12% of the arable land on Earth is flooded (Kaur et al. 2020), which lowers agricultural yields by 10%–50% (Monteleone et al. 2023). The world's WL area is predicted to grow due to ongoing climate change (Xu et al. 2018; Pais et al. 2022). Plant cells underwater have low oxygen content with slow diffusion rates, and the closure of their stomata limits gas exchange. These effects harm the photosynthetic organs of the leaves, lower mesophyll cell activity and chlorophyll content, impair photosynthetic performance, and exacerbate plant chlorosis and membrane lipid peroxidation, which eventually cause the leaves to wither and fall off (Yang et al. 2023). Reduced dry matter synthesis results from a decline in photosynthetic rate, which impacts the development of reproductive organs (Najeeb et al. 2015). WL reduced total protein and soluble sugar content of maize grains (Shao et al. 2023), which is directly related to the developmental stage of the crops (Ren et al. 2023), as well as fewer panicles per plant and grains per panicle in barley (de San Celedonio et al. 2018) and rice (Zhen et al. 2020). WL‐stress also alters the equilibrium between endogenous synthesis and neutralization of reactive oxygen species (ROS), such as superoxide anion radicals and hydrogen peroxide (H_2_O_2_), leading to the buildup of ROS that causes oxidative stress in plants (Anee et al. 2019; Wang et al. 2021). Excess ROS accelerated lipid peroxidation, which resulted in the dysregulation of several physiological systems (Endale et al. 2023). Excessive ROS also disrupts the function of physiological membranes, leading to cell enlargement and rupture (Sarwar et al. 2019).

One of the most popular staple grains in the world is maize ( *Zea mays* L.), which is grown on around 200 million hectares of land (Food and Agriculture Organization 2025; Erenstein et al. 2022). Maize, widely known as corn, was domesticated more than 9000 years ago in southern Mexico and Mesoamerica (Kennett et al. 2020), roughly 10,000 years after wheat was domesticated in the Fertile Crescent of the Near East. It is a more adaptable multipurpose crop when compared to wheat and rice. Most of the maize (70%) is grown in temperate climate; however, it is produced in almost equal proportions in tropical and temperate climates. Maize is regarded as very sensitive to changes in its environment, particularly when it is still a seedling (Wang et al. 2018; Babu et al. 2020). In particular, WL‐stress is quite harmful to maize because it considerably slows down its growth and development during the early growth stage. The biggest growth delay occurs at the seedling stage due to WL (Ren et al. 2014), which also results in withered and yellowed leaves, a reduction in the maximum amount of green leaf area, decreased photosynthetic efficiency, and exacerbated oxidative damage (Wu et al. 2018; Yao 2021). When plants become waterlogged during the seedling stage, as opposed to later stages, grain yield is particularly affected and reduces dramatically. Inadequate drainage in low‐lying areas, in addition to excessive rainfall, is another major factor contributing to WL (Barik et al. 2019). For the production of maize under WL‐stress, increasing resistance to WL is therefore essential.

NO is a widely distributed and highly diffusible gaseous free radical that functions as an intracellular and intercellular messenger in plants, triggering a variety of events by either redox or additive chemistry. As an antioxidant agent, it may scavenge ROS and, as a signaling molecule, modify the expression of antioxidant genes to protect plant cells from oxidative damage (Fan et al. 2014). NO is one of the most important signals during floods. It boosted the activity of the ascorbate peroxidase (APX), antioxidant enzymes catalase (CAT), and superoxide dismutase (SOD) in plants (Faraji and Sepehri 2020). Moreover, it was shown that NO controls respiratory oxygen consumption in pea ( *Pisum sativum* L.) seeds during germination, allowing them to keep some oxygen to avoid experiencing total anoxia (Borisjuk et al. 2007).

GSH is a common and plentiful thiol that serves a variety of purposes in plants. Herbicide detoxification and the transportation of reduced sulfur are involved (Pei et al. 2019). GSH, a significant cellular antioxidant, is thought to be a determinant of cellular redox status, which may have an impact on several essential cellular functions. Additionally, it is thought to be an effective scavenger of ROS and peroxides generated during various stress responses (Hossain et al. 2015). GSH is also necessary for the growth and development of plants (Zechmann et al. 2011). Application of exogenous GSH reduced oxidative stress by increasing the levels of GSH and total flavonoids in the leaf tissues, as well as the activities of glutathione peroxidase, ascorbate peroxidase, peroxidase, catalase, and glutathione *S*‐transferase (GST) (Akbari et al. 2022; Gaafar et al. 2022; Madhu et al. 2023). GSH contributes to thiol redox signaling that is dependent on the glutathione/glutaredoxin system and controls a protein's biological activity in response to physiological, developmental, and environmental stimuli in plants (Pei et al. 2019).

There is currently no research on the relative contributions of individual and combined NO and GSH to the reduction of WL‐stress maize plants. Therefore, the current study aims to assess the effects of both individual and combined GSH and NO under WL situations. Morphophysiological and biochemical techniques were used in combination to accomplish these goals. We measured the following: (1) morphological parameters: shoot lengths, fresh weight and dried weight of the shoot and root; (2) membrane damaging criteria (H_2_O_2_ and MDA); (3) osmolytes (proline in leaf) and soluble sugar and protein; (4) enzymatic antioxidants (POX); (5) nonenzymatic antioxidants and secondary metabolites (ASC and anthocyanins); (6) photosynthesis pigments (Chl *a*, Chl *b*, Chl *a* + *b*, and carotenoids). This research may help elucidate the underlying mechanisms of WL‐stress in maize seedlings and provide valuable insights into the role of NO and GSH in enhancing resistance to WL.

## 2 Materials and Methods

### 2.1 Plant Materials and Experimental Procedure

The maize seeds (BARI hybrid maize‐5) were surface sterilized for 15 min using 2.5% sodium hypochlorite, and then they were washed three times with distilled water. After being sterilized, the seeds were soaked in distilled water and placed in a dark environment for a whole day in order to imbibe. The imbibed seeds were germination‐tested at 25°C ± 2°C in the dark. In plastic pots (volume 350 mL, diameter 10 cm, height 4.5 cm) containing freshly prepared soil, evenly germinated seeds were moved after 48 h and cultivated under control conditions (temperature 25°C ± 2°C, 70% relative humidity). Five treatment groups were established, each containing two sets of pots with seven‐day‐old maize seedlings. One group remained in the control condition, and the remaining four groups were taken to the WL condition. We placed the pots with plants into a large tray (dimensions: length 56.6 × width 30.2 × height 17 cm) and maintained the water level 2 cm above the root zone. The last three groups were treated with foliar spray containing 100 μM NO or/and 200 μM GSH for 5 days. The third, fourth, and fifth pots were treated with only NO, only GSH, and both NO and GSH, simultaneously. The NO and GSH levels were selected based on previous studies (Sohag et al. 2020; Dawood et al. 2023) because these concentrations were recognized as physiologically significant and effective to improve stress tolerance, without any cytotoxic effects. Our experiment included five treatments: C, WL, WL + NO, WL + GSH, and WL + NO + GSH. The same experimental design was applied for three replications of each treatment. After five days of WL‐stress, maize seedlings were collected to assess various morphological, physiological, and biochemical traits.

### 2.2 Determination of Growth Parameters

Five days after WL‐stress, plant roots and stem samples were collected. Initially, measurements were made of the shoot length (SL), shoot fresh weight (SFW), and root fresh weight (RFW). Following 96 h of desiccation at 80°C in a vented oven, the root dry weight (RDW) and shoot dry weight (SDW) of the samples were measured.

### 2.3 Determination of Proline, Soluble Proteins, Soluble Sugars, and Free Amino Acid Contents

Proline and soluble sugar contents were determined using fresh plant leaves. Soluble protein was measured using both fresh leaves and plant roots. The protocol of (Bates et al. 1973) was followed in extracting and determining the proline content. The published approach (Fales 1951) was used to determine the soluble sugar concentration by starting with anthrone‐sulfuric acid. The soluble protein concentration was calculated using the (Lowry et al. 1951) technique, with bovine serum albumin as the reference.

### 2.4 Measurement of Lipid Peroxidation and Hydrogen Peroxide Content

Heath and Packer (1968), outlined the process for determining the degree of lipid peroxidation in terms of MDA. Briefly, 0.2 g of fresh plant sample was homogenized with 5 mL of a 5% trichloroacetic acid (TCA) solution, and the extract was centrifuged at 15,000 *g* for 10 min at 4°C. A 2 mL aliquot of the supernatant was added to 4 mL of a 20% TCA solution containing 0.5% thiobarbituric acid. After 30 min of incubation at 95°C, the mixtures were promptly placed in an ice bath. Following a brief immersion in an ice bath, the supernatant was centrifuged again for 10 min at 11,000 *g*. The absorbance readings were measured at 532 nm. By using the absorption coefficient of 155 nM^−1^ cm^−1^, the MDA content was determined.

With slight adjustments, the technique of Velikova et al. (2000) was used to determine the H_2_O_2_ concentration of leaf tissue. Briefly, after grinding 0.2 g of maize leaves with 1.0 mL of 0.10% TCA solution, the extract was centrifuged at 11,500 *g* for 12 min at 4°C. A test tube was filled with 0.5 mL of the supernatant, 0.5 mL of 10 mM potassium phosphate buffer (pH 7.0), and 1 mL of 1.0 M potassium iodide (KI). The test tubes were then incubated for 60 min in a dark environment. The H_2_O_2_ concentration was expressed as nmol g^−1^ FW, and the absorbance was measured at 390 nm wavelength (Shimadzu, UV‐1201, Kyoto, Japan).

### 2.5 Determination of Ascorbic Acid Content and Guaiacol Peroxidase Activity

The ascorbic acid content (ASA) had been determined using (Jagota and Dani 1982) methodology. A standard curve was created using ASA, and the findings are given as nmol g^−1^ FW. Extracts from the third leaves of maize seedlings were used to measure the activity of antioxidant enzymes. Using pre‐chilled mortars and pestles, 1 mL of 50 mM potassium‐phosphate buffer (pH 8.0) was used to homogenize fresh leaf samples (0.05 g). To examine the activity (guaiacol peroxidase activity) POX (EC: 1.11.1.7), the homogenates were centrifuged at 11,500 × *g* for 10 min, and the resulting supernatants were collected (Nakano and Asada 1981). A UV–Vis spectrophotometer (Shimadzu, UV‐1201, Kyoto, Japan) was used for all spectrophotometric experiments, and all operations were carried out between 0°C and 4°C.

### 2.6 Determination of Anthocyanin Content

In accordance with the methodology of (Abdel‐Aal and Hucl 1999), the absorbance of ethanolic extracts at pH 1.0 was used to measure the total anthocyanin content. A 50 mL centrifuge tube containing 0.5 g of ground leaf material was homogenized with 25 mL of an acid–ethanol solution (0.225 mol equivalent/L HCl in ethanol‐water (95:5, v/v)). Supernatants were collected after the tube was centrifuged at 3000 × *g* (Sorvall RC5C, Sorvall Instruments, Dupont, Wilmington, DE) for 15 min after being flushed with nitrogen gas and shaken for 30 min. In a photodiode array spectrophotometer, absorbance measurements at 535 nm were adjusted for background absorbance at 700 nm (caused by turbidity). Using a molecular weight of 449.2 g/mol and a molar extinction value of 25,965 M^−1^ cm^−1^, anthocyanins were expressed as mg of cyanidin‐3 glucoside equivalents/100 g (Abdel‐Aal and Hucl 1999).

### 2.7 Determination of Leaf Chlorophyll and Carotenoid Contents

A spectrophotometer (Shimadzu, UV‐1201, Kyoto, Japan) was used to assess the total photosynthetic pigment, or total chlorophyll content, of maize seedlings. The procedure outlined by (Metzner et al. 1965) was followed with a few minor adjustments. To extract the pigments, 10 mL of 80% (v/v) aqueous acetone was applied to a 0.5 g sample in a screw‐capped tube, which was then left for seven days. A spectrophotometric absorbance measurement at 644 and 663 nm wavelengths was performed using the supernatant after the plant acetone extract was centrifuged for 10 min at 4000 × *g*. The chlorophyll contents were calculated using the following formulas, and the total chlorophyll content was calculated as the sum of the chlorophyll *a* and *b* contents and reported as mg g^−1^ fresh weight (FW). Carotenoids were extracted and determined using the published technique (Lichtenthaler 1987).

### 2.8 Statistical Analysis

All statistical analysis and figure preparation were performed using R version 4.4.2. The package “*multcomp*” was used to perform two‐way analysis of variance and Tukey's test (*p* < 0.05). The figures were generated using the package “*ggplot2*”. The heatmap was performed using “*pheatmap*” and the principal component analysis was performed using “*FactoMineR*” and “*Factoextra*” packages. Means ± standard errors (SEs) of three independent replications for each treatment are shown in the figures.

## 3 Results

### 3.1 Effects of Exogenous GSH and NO on Phenotypic Appearances of Maize Under WL‐Stress

**FIGURE 1:**
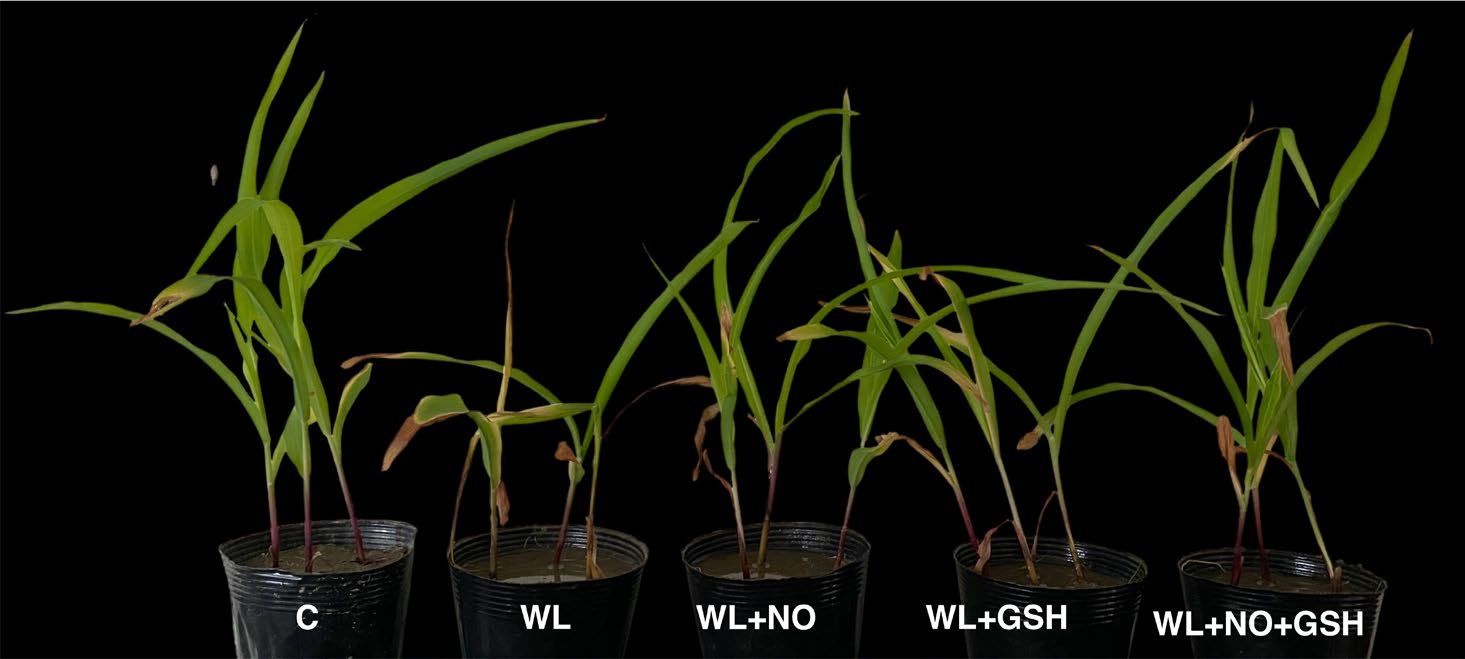
Phenotypic appearance of nitric oxide (NO)‐ and/or glutathione (GSH)‐treated maize seedlings grown under WL‐stress. C, control (no treatment); WL, waterlogging; NO, 100 μM NO; GSH, 200 μM GSH; NO + GSH, 100 μM NO+ 200 μM GSH.

Under WL‐stress, maize seedlings demonstrated substantial morphological abnormalities, as evidenced by several toxic signs, including chlorosis, necrosis, and rolling of the leaves. Exogenous NO and GSH pretreatments decreased the symptoms of WL‐stress damage, and NO + GSH had a significant impact on plants under WL‐stress (Figure 1).

### 3.2 Effects of Exogenous GSH and NO on Growth of Maize Under WL‐Stress

**FIGURE 2:**
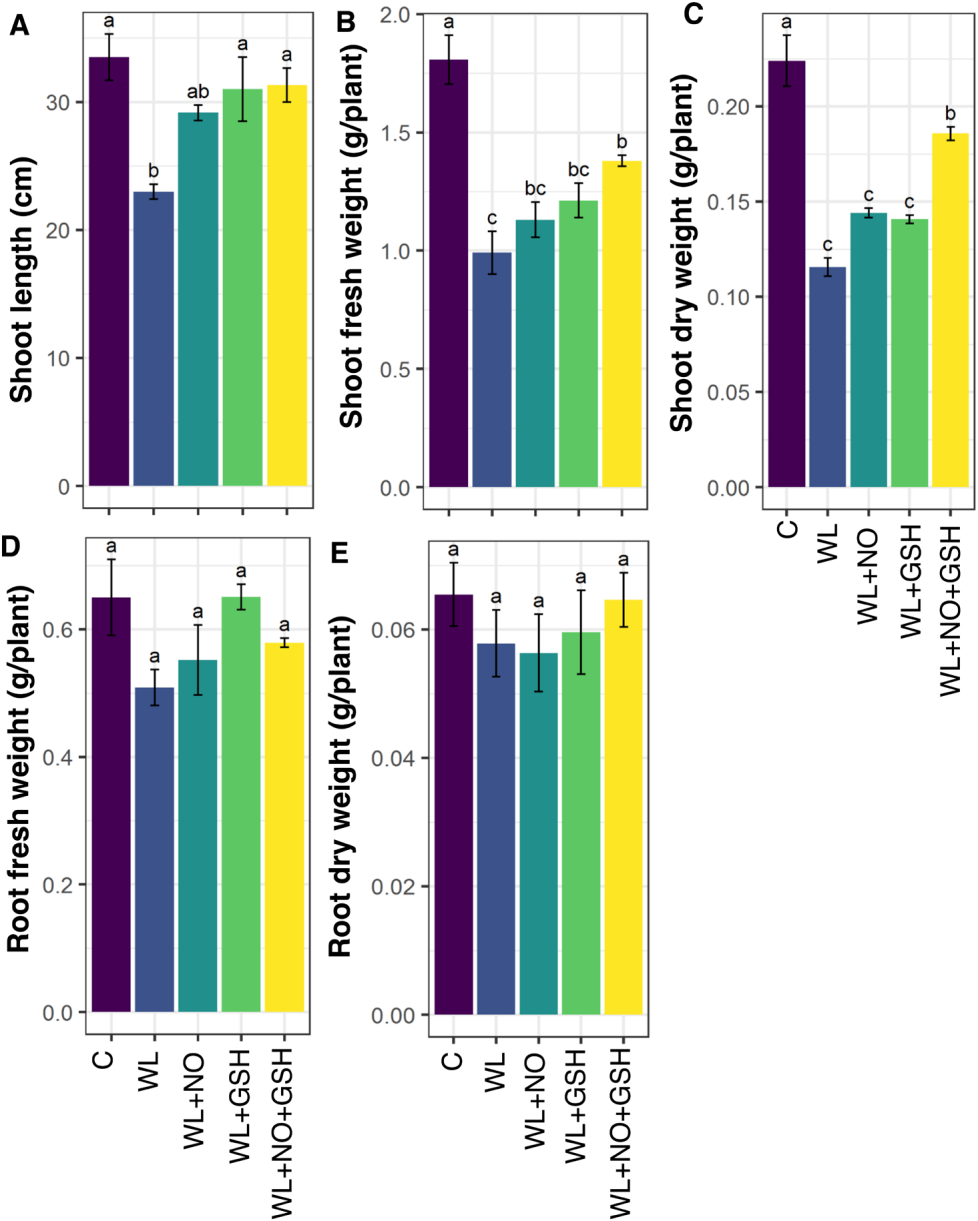
Effects of exogenous nitric oxide (NO) and glutathione (GSH) pretreatments on (A) shoot length (SL), (B) shoot fresh weight (SFW), (C) shoot dry weight (SDW), (D) root fresh weight (RFW), and (E) root dry weight (RDW) of maize seedlings grown under normal or waterlogging condition for 5 days. Data represent means of three independent replicates (*n* = 3). Vertical bars indicate standard errors. Different letters represent significant differences at *p* < 0.05, based on Turkey's test. C, control (no treatment); WL, waterlogging; NO, 100 μM NO; GSH, 200 μM GSH; NO + GSH, 100 μM NO + 200 μM GSH.

The maize seedlings exposed to WL‐stress showed a significant reduction in SL, SFW, and SDW compared to non‐stressed seedlings (Figure 2). When WL‐maize seedlings were treated with GSH or NO, they exhibited better shoot growth, length, and biomass than only WL‐stressed seedlings (Figure 2). However, only GSH‐treated seedlings showed significantly higher SL compared to only WL‐stressed plants. Moreover, the combined application of GSH and NO in WL‐stressed seedlings significantly improved SL compared to only WL‐stressed seedlings (Figure 2). In the case of root fresh and dry weight, no significant differences were found across the treatment conditions.

### 3.3 Effects of Exogenous GSH and NO on H_2_O_2_ and MDA Content of Maize Under WL‐Stress

**FIGURE 3:**
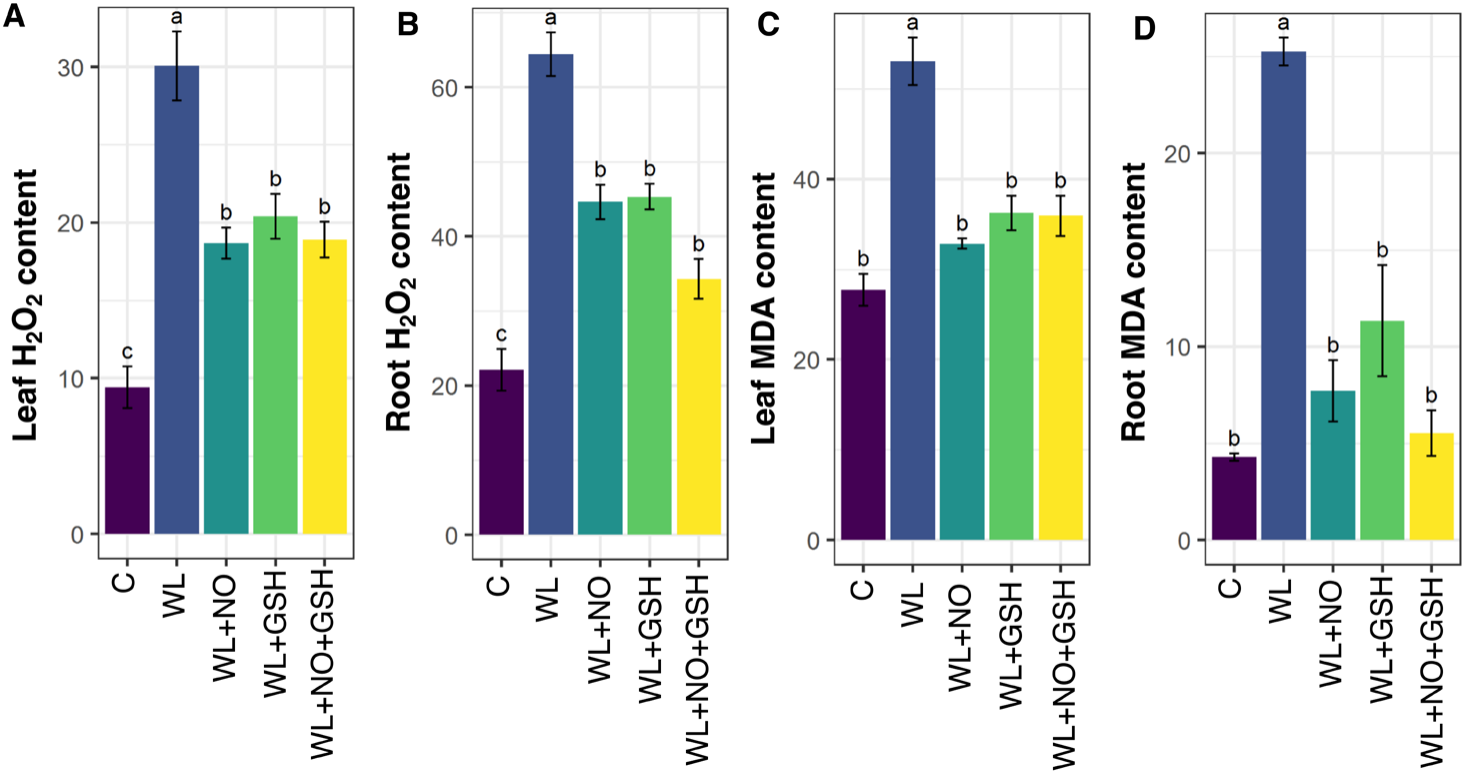
Effects of exogenous nitric oxide (NO) and glutathione (GSH) pretreatments on (A) Leaf H_2_O_2_ content (nmol g^−1^ FW), (B) Root H_2_O_2_ content (nmol g^−1^ FW), (C) Leaf MDA content (nmol g^−1^ FW), (D) Root MDA content (nmol g^−1^ FW) of maize seedlings grown under normal or WL‐stress condition for 5 days. Data represent means of three independent replicates (*n* = 3). Vertical bars indicate standard errors. Different letters represent significant differences at *p* < 0.05, based on Turkey's test. C, control (no treatment); WL, waterlogging; NO, 100 μM NO; GSH, 200 μM GSH; NO + GSH, 100 μM NO + 200 μM GSH.

Both leaf and root H_2_O_2_ content were significantly higher under WL conditions than in the control conditions (Figure 3). When maize seedlings were treated with GSH, NO, or both, they exhibited significantly lower content of H_2_O_2_ compared to WL‐stressed seedlings.

Same as H_2_O_2_ content in leaf and root, leaf and root MDA content was found significantly higher in WL‐stressed seedlings than in control seedlings. On the other hand, NO‐, GSH‐, and NO + GSH‐treated WL‐seedlings showed significantly lower MDA content in both root and leaf than only WL‐stressed seedlings (Figure 3).

### 3.4 Effects of Exogenous GSH and NO on Soluble Sugar, Proline, and Protein Content of Maize Under WL‐Stress

**FIGURE 4:**
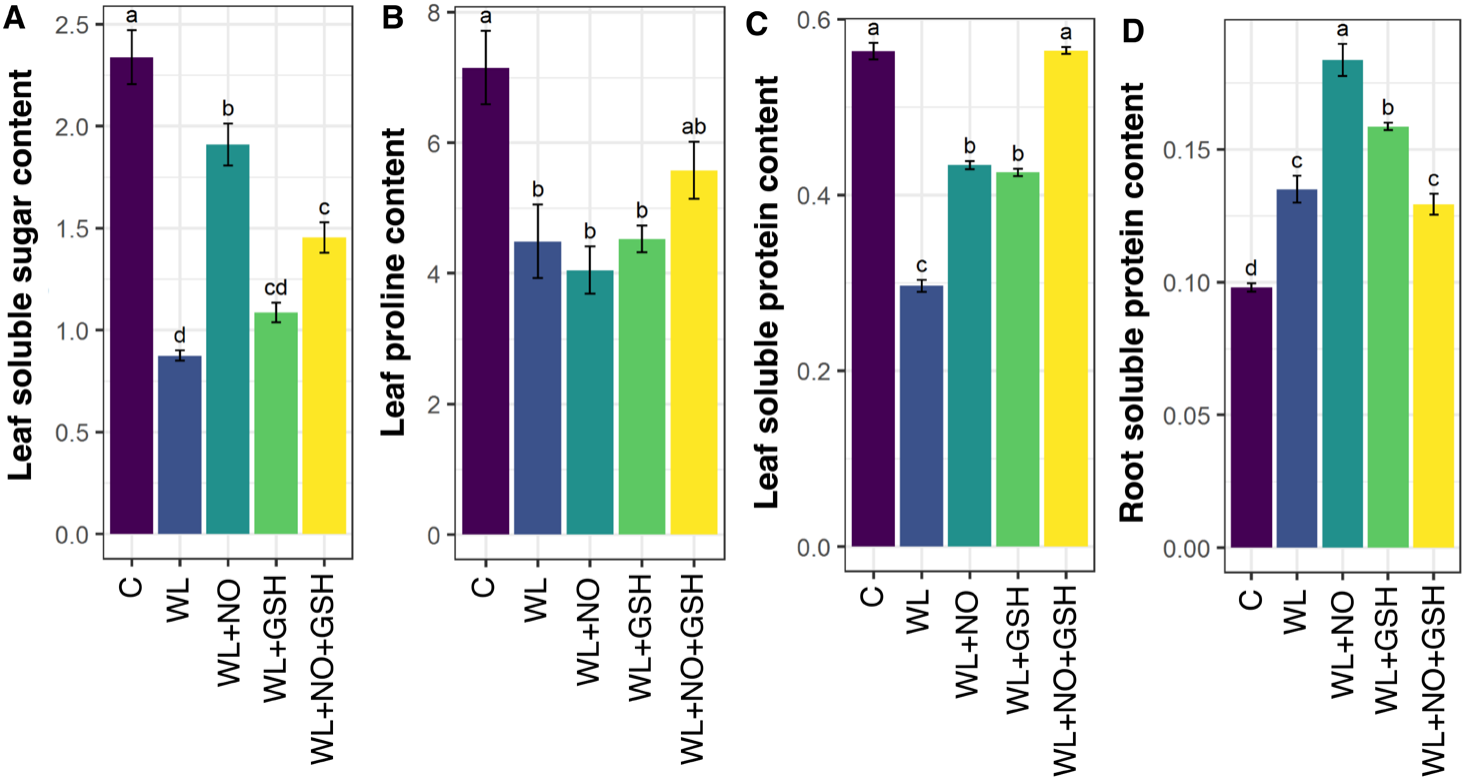
Effects of exogenous nitric oxide (NO) and glutathione (GSH) pretreatments on (A) Leaf soluble sugar content (mg g^−1^ FW), (B) Leaf proline content (μg g^−1^ FW), (C) Leaf soluble protein content (mg g^−1^ FW), (D) Root soluble protein content (mg g^−1^ FW) of maize seedlings grown under normal or waterlogging condition for 5 days. Data represent means of three independent replicates (*n* = 3). Vertical bars indicate standard errors. Different letters represent significant differences at *p* < 0.05, based on Turkey's test. C, control (no treatment); WL, waterlogging; NO, 100 μM NO; GSH, 200 μM GSH; NO + GSH, 100 μM NO + 200 μM GSH.

WL‐stressed seedlings showed significantly lower soluble sugar and proline content in the leaf compared to normal seedlings. However, the application of NO and NO + GSH in WL‐stressed seedlings significantly improved the content of soluble sugar in the leaf. On the other hand, NO and GSH‐treated WL‐stressed seedlings showed higher proline content than only WL‐stressed seedlings (Figure 4A,B).

WL‐stress in maize significantly reduces the leaf‐soluble protein and increases the root‐soluble protein compared to normal seedlings. When WL‐maize seedlings were treated with GSH or NO exhibited better soluble protein in the leaf than only WL‐stressed seedlings (Figure 4C,D). Moreover, NO + GSH‐treated seedlings showed significantly higher soluble protein in the leaf than only WL‐stressed plants (Figure 4C). The higher soluble protein content was found in the root of NO‐treated and GSH‐treated WL‐stressed seedlings compared to only WL‐stressed seedlings (Figure 4D).

### 3.5 Effects of Exogenous GSH and NO on ASC, POX, and Anthocyanin Content in Maize Under WL‐Stress

**FIGURE 5:**
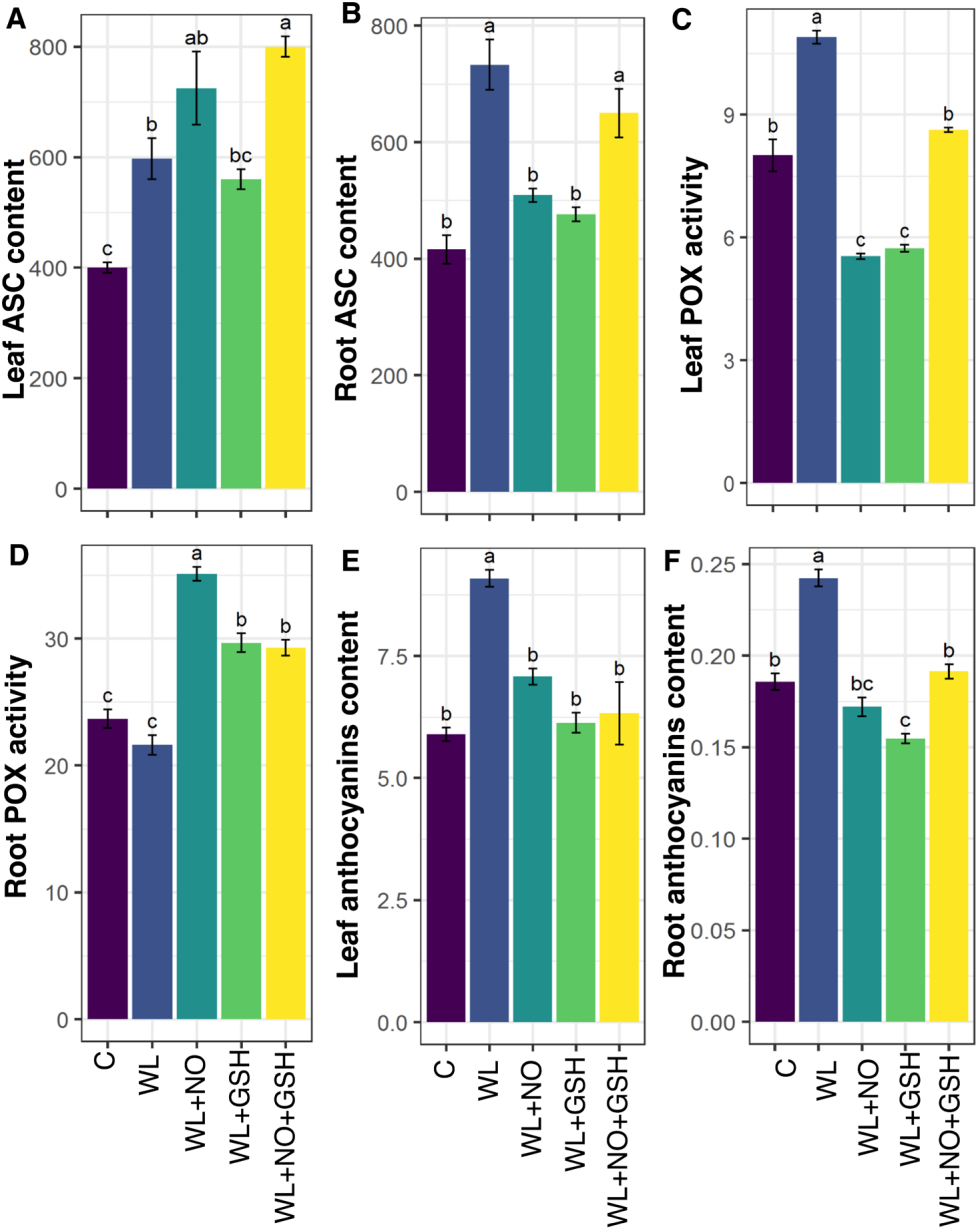
Effects of exogenous nitric oxide (NO) and glutathione (GSH) pretreatments on (A) Leaf ASC content (μg g^−1^ FW), (B) Root ASC content (μg g^−1^ FW), (C) Leaf POX activity (μmol min^−1^ g^−1^ FW), (D) Root POX activity (μmol min^−1^ g^−1^ FW), (E) Leaf anthocyanins content (μg g^−1^ FW), (F) Root anthocyanins content (μg g^−1^ FW) of maize seedlings grown under normal or waterlogging condition for 5 days. Data represent means of three independent replicates (*n* = 3). Vertical bars indicate standard errors. Different letters represent significant differences at *p* < 0.05, based on the Turkey's test. C, control (no treatment); WL, waterlogging; NO, 100 μM NO; GSH, 200 μM GSH; NO + GSH, 100 μM NO + 200 μM GSH.

Leaf and root ASC contents were significantly higher in WL‐stressed seedlings compared to non‐stressed seedlings. Moreover, NO + GSH‐treated WL‐stressed seedlings exhibited significantly higher ASC contents in root and leaf than non‐stressed seedlings. However, when WL‐stressed maize seedlings were treated with NO or GSH individually, they showed significantly lower ASC in root compared to WL‐stressed seedlings (Figure 5A,B).

Leaf POX activity was significantly higher in WL‐stressed seedlings than in non‐stressed seedlings. NO + GSH‐treated WL‐stressed seedlings showed significantly lower POX in leaf than WL‐stressed seedlings. Moreover, when NO or GSH was individually applied to WL‐stressed seedlings, the POX activity of leaf decreased significantly compared to that of non‐stressed seedlings (Figure 5C). On the other hand, root POX activity is significantly high in NO‐, GSH‐, and NO + GSH‐treated plants compared to WL‐stressed seedlings (Figure 5D).

Higher anthocyanin content was found in both leaf and root of maize seedlings under WL‐stress than in non‐stress conditions. NO‐, GSH‐, and NO + GSH‐treated WL‐stressed seedlings showed significantly lower anthocyanin content than only WL‐stressed seedlings (Figure 5E,F).

### 3.6 Effects of Exogenous GSH and NO on Photosynthetic Pigments of Maize Under WL‐Stress

**FIGURE 6:**
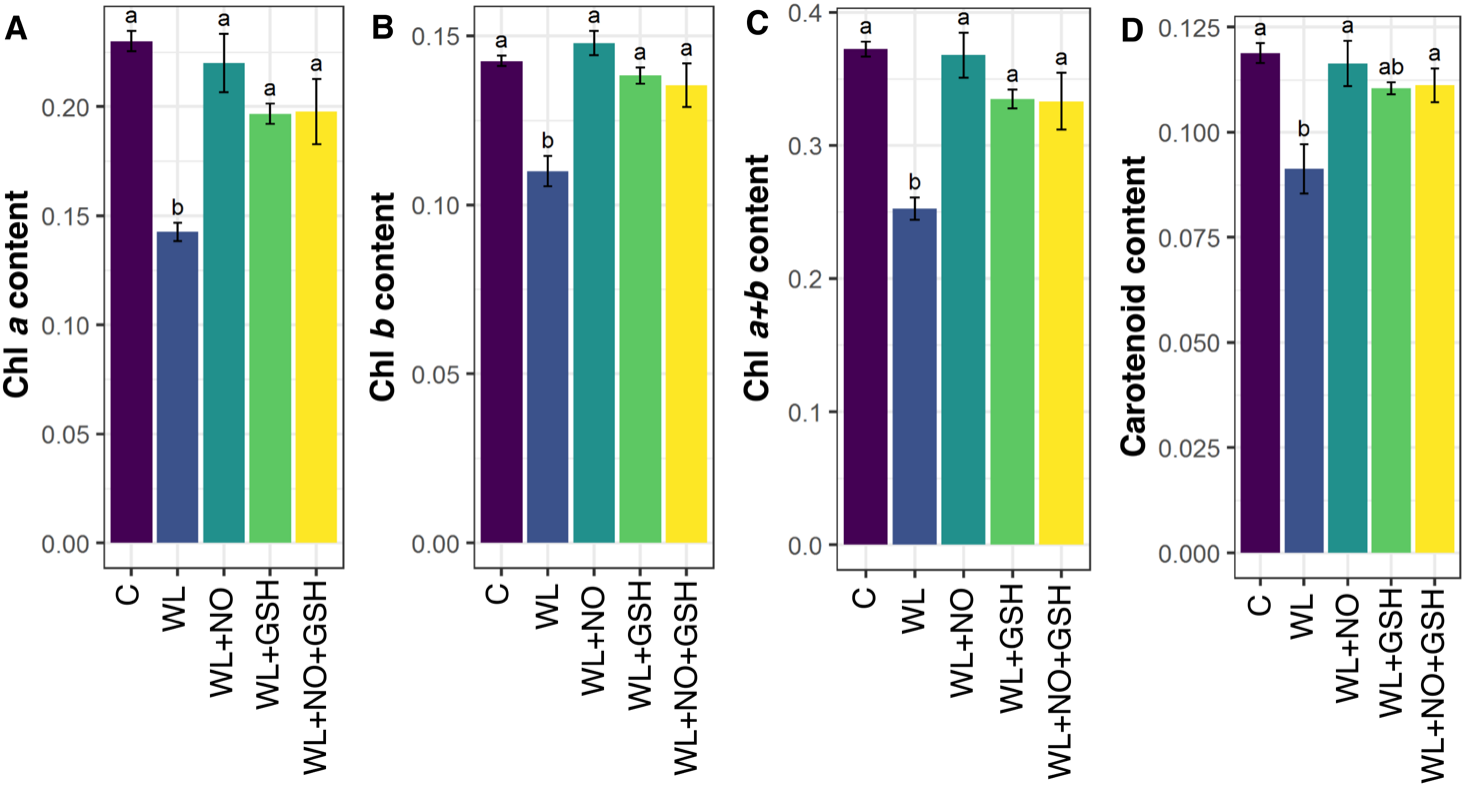
Effects of exogenous nitric oxide (NO) and glutathione (GSH) pretreatments on (A) Chlorophyll (Chl) *a* content (mg g^−1^ FW), (B) Chl *b* content (mg g^−1^ FW), (C) Chl *a + b* content (mg g^−1^ FW), (D) carotenoid content (mg g^−1^ FW) of maize seedlings grown under normal or waterlogging condition for 5 days. Data represent means of three independent replicates (*n* = 3). Vertical bars indicate standard errors. Different letters represent significant differences at *p* < 0.05, based on the Turkey's test. C, control (no treatment); WL, waterlogging; NO, 100 μM NO; GSH, 200 μM GSH; NO + GSH, 100 μM NO + 200 μM GSH.

The maize seedlings exposed to WL‐stress showed a significant reduction in Chl *a*, Chl *b*, Chl *a + b*, and carotenoid contents compared to non‐stressed seedlings (Figure 6A–D). When WL‐stressed maize seedlings were treated with GSH or NO or GSH + NO, they exhibited significantly higher Chl *a*, Chl *b*, Chl *a + b*, and carotenoid contents than only WL‐stressed seedlings (Figure 6A–D).

### 3.7 Heat Map and PCA Analyses

**FIGURE 7:**
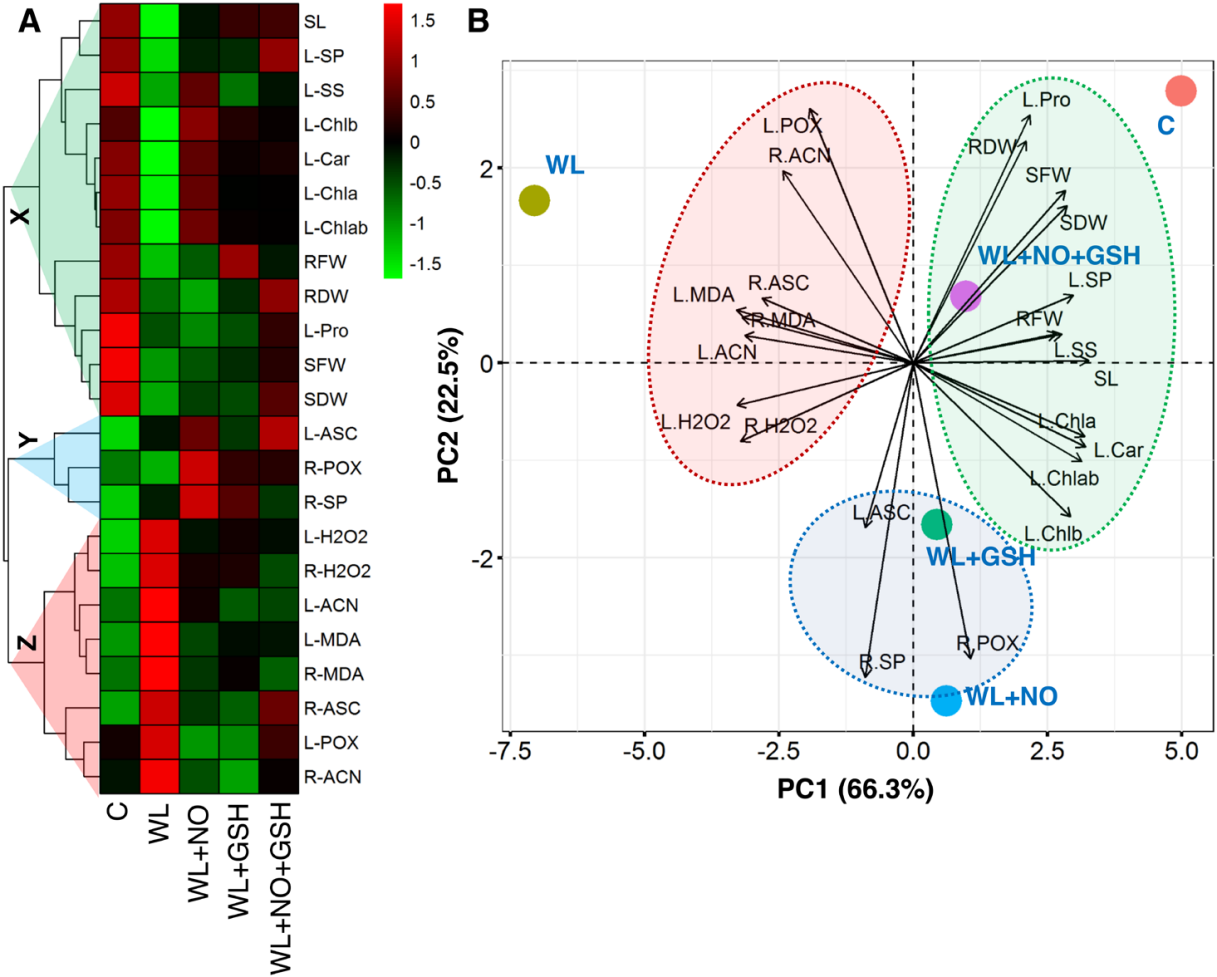
Hierarchical clustering with heatmap of studied parameters of leaf (L) and root (R) (A) and principal component analysis (PCA) of studied parameters of leaf (L) and root (R) (B) of maize seedlings. The variables included shoot length (SL), shoot fresh weight (SFW), shoot dry weight (SDW), root fresh weight (RFW), root dry weight (RDW), chlorophyll (chl), carotenoid (Car), proline (Pro), hydrogen peroxide (H_2_O_2_), malondialdehyde (MDA), anthocyanins (ACN), soluble sugar (SS), soluble protein (SP), ascorbic acid (ASC), and peroxidase (POX).

According to the cluster heatmap, a total of 23 traits were arranged in three clusters (cluster‐X, Y, and Z). Cluster‐X contained the largest number of traits, such as SL, L‐SP, L‐SS, L‐Chl b, L‐Car, L‐Chl a, L‐Chlab (Chl *a + b*), RFW, RDW, L‐Pro, SFW, and SDW, which were higher in control and WL + NO + GSH treatments (Figure 7A). The cluster‐Z comprised L‐H_2_O_2_, R‐H_2_O_2_, L‐ACN, R‐MDA, L‐MDA, R‐ASC, R‐ACN, and L‐POX, which were higher in WL treatment (Figure 7A).

The PC1 and PC2 explained a total of 88.80% of the variability. PCA showed that the parameters of cluster‐X are strongly and positively correlated with the C and WL + NO + GSH treatments. Moreover, the WL treatments showed a strong and positive correlation with the parameters of cluster‐Z (Figure 7B).

## 4 Discussion

Very few studies have examined the effect of both NO and GSH on plant abiotic stress response. Exogenous NO and GSH have the potential to mitigate heavy metal stress (Mostofa et al. 2015); however, there is no study on the mitigation process of WL‐stress. Several studies have examined the individual effects of GSH (Cao et al. 2017; Banerjee and Roychoudhury 2019; Pei et al. 2019; Koh et al. 2021; Keya et al. 2022) and NO (Fancy et al. 2017; Khan et al. 2019; Keya et al. 2022; Praveen 2022) on mitigating the impacts of heavy metals, drought, salinity, WL, and extreme temperatures (both heat and cold) stresses. Exogenous GSH improves antioxidant defense, controls ionic and osmotic homeostasis, and upregulates stress‐responsive genes successfully to reduce drought, salt, and Cd stress in maize (Pei et al. 2019). Moreover, NO is a necessary signaling molecule for controlling plant growth and development (Singh et al. 2020).

### 4.1 Do Exogenous GSH and NO Improve WL‐Stress Tolerance in Maize?

The beneficial effects of exogenous GSH and NO on WL‐stress tolerance in maize have not been investigated extensively. In the current study, those molecules were foliar‐sprayed on the leaves of the plants to investigate their effects on the tolerance and survival of maize seedlings to short‐term WL circumstances. After five days of WL‐stress exposure, maize seedlings showed several phenotypic abnormalities, including stunted growth, decreased survival, and shoot biomass and root biomass reduction (Figures 1 and 2A–D). However, WL‐stressed maize seedlings that received foliar applications of GSH and NO alone and in combination were able to partially or fully recover from all of the previously indicated adverse effects (Figures 1 and 2A–E). According to some research, rice (Siddiqui et al. 2021) and tomato ( *Solanum lycopersicum* ) (Yan et al. 2018; Zhou et al. 2019) both showed GSH‐mediated active growth recovery from salinity and submergence stresses, respectively. The majority of plants are susceptible to WL‐stress because it greatly reduces photosynthesis and respiration, as well as the rates at which O_2_ and CO_2_ diffuse through the roots and stems of the plant (Pan et al. 2021). However, this study shows that short‐term WL‐stress did not alter the root dry and fresh weight in maize plants (Figure 3D,F).

Waterlogging leads to low O_2_ levels in plant tissues, which can result in an overproduction of ROS due to the imbalance between ROS generation and detoxification (Anee et al. 2019; Park and Lee 2019). The initial ROS generated is typically superoxide, which then spontaneously forms H_2_O_2_ through dismutation (Wang et al. 2021). The excessive production of H_2_O_2_ in waterlogged plants can negatively impact various physiological processes because H_2_O_2_ is a potent, uncharged oxidant molecule (Castro‐Duque et al. 2020). The higher MDA concentration represented the ROS‐mediated membrane damage (Ashraf 2012). The addition of exogenous GSH and NO resulted in a considerable reduction in the amount of H_2_O_2_ (Figure 2A,B) and MDA (Figure 3C,D) in both leaf and root in waterlogged maize plants compared to the non‐stressed plants.

### 4.2 How Do Exogenous GSH and NO Improve WL‐Induced Oxidative Stress Tolerance in Maize?

Ascorbic acid (ASC) is a main component of the plant antioxidant system that functions both as a direct scavenger of ROS and as a key redox buffer in the ascorbate‐glutathione (ASC‐GSH) cycle (Bilska et al. 2019). It is a vital redox buffer and cofactor for enzymes that control photosynthesis, hormone production, and the regeneration of other antioxidants. It also controls cell division and proliferation and participates in signal transmission (Gallie 2013). WL‐stress disrupts mitochondrial respiration and cellular redox homeostasis, which leads to excessive accumulation of ROS such as H_2_O_2_ and superoxide radicals. The significant increase in ASC content was observed in both leaf and root under WL‐stressed maize seedlings (Figure 5A,B). In WL conditions, maize plants auto‐generate the ASC, which partially helps the plants to stand in WL‐stress. A similar result has been reported in maize and other crops, where increased ASC accumulation helps maintain cellular redox balance and protect membrane integrity under WL‐stressed conditions (Zhang et al. 2021; Pan et al. 2021). Notably, NO + GSH‐treated WL‐stressed seedlings exhibited even higher ASC levels than non‐stressed plants, which indicates a combined interaction between NO and GSH in promoting ASC accumulation. This response is explained by the ability of NO to modulate redox signaling and upregulate antioxidant‐related genes, while GSH serves as a direct electron donor for dehydroascorbate (DHA) reduction via dehydroascorbate reductase, thereby sustaining ASC pools (Foyer and Noctor 2011; Hasanuzzaman et al. 2022). In contrast, the significantly lower root ASC content was observed in WL‐stressed seedlings treated with NO or GSH alone. It suggests that individual application of either molecule was insufficient to maintain ASC homeostasis under WL stress. NO alone promotes ASC oxidation or redirects redox signaling toward nitrosative pathways; on the other hand, GSH alone may be preferentially consumed in ROS detoxification without adequate regeneration of ASC, leading to reduced ASC pools in roots. Our results show that ASC accumulation resulting from WL stress is not simply a passive stress response but is influenced by redox regulation through the self‐regulated function of NO‐GSH.

Waterlogging generally damages the protective enzyme system by reducing the activity of POX and increasing the content of MDA. This suggests that WL‐stress affects the integrity and peroxidation of membrane lipids, which in turn causes membrane deterioration and accelerates leaf senescence (Ren et al. 2016). In our study, leaf POX activity increased under WL conditions, but individual NO and GSH treatments decreased POX content in the leaves of maize seedlings. POX activity in the root under WL‐stress was similar to the control condition (Figure 5D), but increased when applying NO in the WL‐stress condition. Exogenous NO treatment, such as sodium nitroprusside (SNP), not only boosted the development of maize seedlings under stress circumstances but also increased antioxidant activities, including peroxidase (POX), catalase, and SOD, reducing lipid peroxidation and electrolyte leaks (Pandey et al. 2023).

The anthocyanin index indicates stress‐protective components that plants generate during stress (Qin et al. 2021; Beegum et al. 2023). Higher anthocyanin content in the leaf and root is shown in the WL‐stress condition compared to the control condition (Figure 5E,F). Anthocyanin reduced oxygen levels in plant tissues, as well as nutritional deficiencies or imbalances, influencing photosynthetic and transpiration processes (Beegum et al. 2023).

### 4.3 Do Exogenous GSH and NO Protect Photosynthetic Pigments Under WL‐Stress?

Chlorophyll *a*, *b*, *a + b*, and carotenoid contents in maize seedlings were considerably decreased under WL‐stress (Figure 6A–D) due to increasing leaf chlorosis; as a result, the number of chlorophylls is reduced (Avivi et al. 2020). WL also reduces soluble protein concentration, impacting carbon assimilation, and destroys chlorophyll, leading to decreased photoassimilation (Huang et al. 2022). Under WL‐stress situations, maize leaves must close their stomata, reduce transpiration and photosynthetic rates, and wilt their blades (Zhang, Huang, et al. 2023). With the extension of waterlogged time, chlorophyll concentration, the associated photosynthetic enzymes, and PSII photochemical efficiency were lowered, leading to a considerable yield drop (Sharma et al. 2022). Treated with NO, GSH, or NO + GSH in WL‐stressed maize seedlings, significantly higher chlorophyll *a*, chlorophyll *b*, chlorophyll *a* + *b*, and carotenoid contents were observed (Figure 6A–D), indicating that these treatments effectively mitigated WL‐induced damage to the photosynthetic machinery.

NO serves as a redox signaling molecule that safeguards chloroplast ultrastructure and stabilizes photosystem II by modulating ROS homeostasis and preventing oxidative chlorophyll breakdown. NO has been demonstrated to augment chlorophyll production by regulating the expression and activity of critical enzymes, including δ‐aminolevulinic acid dehydratase and protochlorophyllide reductase, in response to abiotic stress conditions (Zhang et al. 2022; Suliman et al. 2024). Furthermore, GSH is pivotal in preserving chlorophyll stability during WL stress by detoxifying ROS via the ascorbate–glutathione cycle and mitigating membrane lipid peroxidation, as seen by reduced malondialdehyde (MDA) levels. Reduced lipid peroxidation maintains thylakoid membrane integrity, crucial for the stability of pigment–protein complexes and light‐harvesting efficiency (Sohag et al. 2020). Furthermore, GSH enhances chlorophyll biosynthesis by promoting the absorption and intracellular availability of divalent cations like Mg^2+^, an essential structural element of the chlorophyll molecule, thus aiding continuous chlorophyll production under stress conditions (Sohag et al. 2020).

### 4.4 How Do Exogenous GSH and NO Improve Primary Metabolites in Maize WL‐Stress?

In our study, WL stress resulted in lower leaf soluble sugar content compared to the general condition (Figure 4A). Individual NO and combined GSH and NO treatments on WL‐stress can increase the soluble sugar content. NO increases the expression and activity of enzymes involved in sugar production pathways, such as sucrose synthase and invertase, resulting in the buildup of soluble sugars in plant tissues. The combined effect of NO and GSH may improve the activity of sugar metabolism enzymes while shielding them from oxidative damage, resulting in a greater rise in soluble sugar content than solo therapies.

Proline also functions as an antioxidant, shielding cells from free radical damage and preserving the cell surroundings for the optimal production of biomolecules involved in stress adaptation (Ghosh et al. 2022). Most previous investigations have found that proline content increases with WL‐stress (Bajpai and Chandra 2015; Chávez‐Arias et al. 2019). However, in our findings, the amount of leaf proline decreased in WL stress (Figure 4B) because of inhibition of proline biosynthesis and enhanced catabolism, particularly due to suppressed P5CS activity and stimulation of proline dehydrogenase under hypoxia, which is validated by some other research (Barickman et al. 2019; Hasanuzzaman et al. 2022; Zhang, Huang, et al. 2023).

In WL‐stressed seedlings, exogenous NO and GSH treatments significantly raised proline and soluble sugar levels, probably by controlling osmolyte metabolism and reestablishing redox equilibrium. While GSH preserves cellular redox equilibrium necessary for osmolyte synthesis, NO is known to increase proline buildup by boosting P5CS (Delta‐1‐pyrroline‐5‐carboxylate) expression and inhibiting proline degradation (Ghosh et al. 2022; Hasanuzzaman et al. 2022). These findings suggest that WL‐induced metabolic dysfunction in maize leaves is somewhat mitigated by NO‐ and GSH‐mediated redox control.

Under WL stress, leaf soluble protein content decreased (Figure 4C). This condition influences carbon assimilation by degrading chlorophyll (Ma et al. 2022), which leads to a decrease in photo‐assimilation. Under WL circumstances, maize leaves experience stomatal closure, decreased transpiration and photosynthetic rates, and leaf blade wilting (Qi et al. 2021; Tian et al. 2021). With increased WL‐stress duration, chlorophyll concentration, associated photosynthetic enzymes, and PSII photochemical efficiency were lowered, leading to a considerable production drop (Zhang, Yue, et al. 2023). Soluble Protein content can be increased by the treatment with NO and GSH. GSH protects proteins from oxidative damage, retaining their integrity and avoiding degradation. Enhancing antioxidant defenses helps to preserve protein production and stability under such conditions (Sharma et al. 2019). NO therapy influences protein synthesis pathways, which helps to enhance soluble protein content (Khan et al. 2015). GSH and NO treatment together on WL‐stress in maize has not been studied before. They may work together to modify leaf‐soluble protein concentration under stress since they both engage in antioxidant defense mechanisms and signaling pathways that impact protein metabolism. Plant proteins serve a variety of enzymatic, structural, and functional activities. They also serve as storage media for seedlings, meeting their development and nutritional requirements (Rasheed et al. 2020). Root soluble protein was higher under WL stress than in the control condition. NO and GSH also play a great role in increasing the soluble protein in plant roots (Figure 4D).

## 5 Conclusions

**FIGURE 8:**
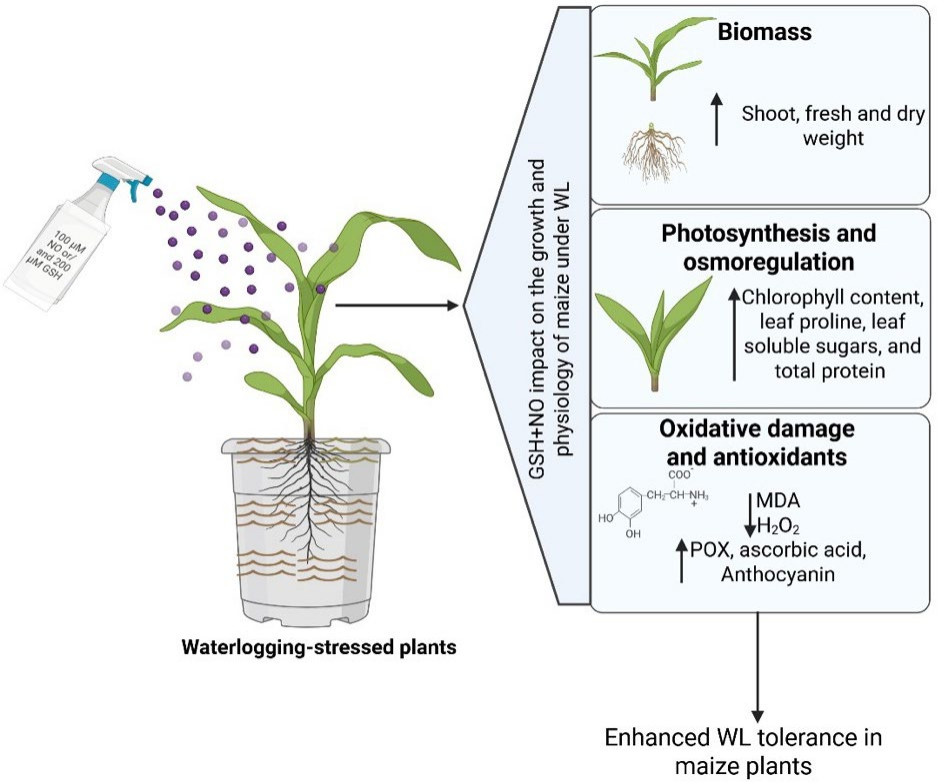
Exogenous application of glutathione (GSH) and nitric oxide (NO) enhances waterlogging (WL) tolerance in maize. Waterlogging‐stressed maize plants treated with 100 μM NO and 200 μM GSH showed improved growth and physiological responses. GSH + NO application enhanced biomass accumulation (shoot and root fresh/dry weight), increased photosynthetic pigments and osmoregulatory molecules (chlorophylls, proline, soluble sugars, total protein), and reduced oxidative damage (lower MDA and H_2_O_2_) by upregulating antioxidant defenses (peroxidase, ascorbic acid, and anthocyanins). These changes contributed to improved WL tolerance in maize plants.

The present study demonstrates that WL stress severely impairs maize seedling physiology by reducing soluble sugar, proline, and protein contents; increasing ROS accumulation and oxidative damage; disrupting photosynthetic pigments; and ultimately suppressing growth (Figure 8). Exogenous application of GSH and NO, particularly in combination, significantly mitigates these adverse effects by enhancing antioxidant enzyme activities, improving photosynthetic pigment levels, and reducing lipid peroxidation, thereby promoting overall seedling vigor (Figure 8). The synergistic effect of GSH and NO in alleviating WL‐induced oxidative stress highlights their potential as effective agents for improving WL tolerance in maize. However, while these findings provide valuable insights into the physiological mechanisms involved, they are based on controlled conditions. Future studies should therefore focus on field‐based evaluations across diverse agroecological zones to validate and optimize the practical application of GSH and NO in maize crop management under waterlogged environments.

## Funding

The authors have nothing to report.

## Conflicts of Interest

The authors declare no conflicts of interest.

## Data Availability

The data that support the findings of this study are openly available in “figshare” at https://doi.org/10.6084/m9.figshare.30901697.
